# Autoantibody against aquaporin-5 may be a new diagnostic biomarker for primary Sjögren’s syndrome

**DOI:** 10.1007/s10067-024-07190-1

**Published:** 2024-10-23

**Authors:** Xiaoyu Wang, Hong Wu, Bing Zhong, Ligai Zhang, Yong Wang

**Affiliations:** 1 Department of Rheumatology and Immunology, Southwest Hospital, Army Medical University (the Third Military Medical University), Chongqing, 400038 People’s Republic of China; 2 Department of Clinical Laboratory, Southwest Hospital, Army Medical University (the Third Military Medical University), Chongqing, 400038 People’s Republic of China

**Keywords:** Anti-aquaporin autoantibody, Biomarker, Primary Sjögren’s syndrome

## Abstract

The study aims to assess the diagnostic and clinical significance of autoantibodies against aquaporin-1 (anti-AQP1) and aquaporin-5 (anti-AQP5) in primary Sjögren’s syndrome (pSS). A total of 163 participants were categorized into three groups: pSS group, other connective tissue diseases (CTD) group, and healthy control (HC) group. The levels of anti-AQP1 and anti-AQP5 autoantibodies in serum were determined using enzyme-linked immunosorbent assay (ELISA), and clinical data from patients were collected for statistical analysis. Our results showed that the level of anti-AQP1 in the pSS group was higher than in the HC group (*P* < 0.05), and no significant difference was observed between the pSS group and the CTD group (*P* > 0.05). ROC showed that the anti-AQP1 had no diagnostic value for pSS (*P* > 0.05). The anti-AQP5 level of 39 healthy adults was all below the cut-off value (14.10 ng/ml) (*P* < 0.05). The level of anti-AQP5 in the pSS group was higher than the CTD group (*P* < 0.05), the AUC was 0.86 (95% CI 0.80–0.93), with a sensitivity of 0.95 (95% CI 0.87–0.99) and a specificity of 0.70 (95% CI 0.58–0.84). No correlation was found between anti-AQP5 levels and the EULAR primary Sjögren’s syndrome disease activity index score, anti-SSA, anti-SSB, antinuclear antibodies, rheumatoid factor, anti-ds-DNA, salivary gland flow rate, complement 3, and lymphocyte count in pSS samples (*P* > 0.05), respectively. Therefore, the elevated anti-AQP5 may emerge as a novel diagnostic biomarker for pSS patients due to high sensitivity and specificity.

**Key Points**

*• The elevated anti-AQP5 may emerge as a novel diagnostic biomarker for pSS patients due to high sensitivity and specificity.*

## Introduction

Primary Sjögren’s syndrome (pSS) is a persistent autoimmune condition marked by substantial lymphocyte infiltration into exocrine glands, leading to the partial destruction of exocrine gland tissue and diminished gland function. The primary clinical indicators encompass dry mouth and dry eyes, and in certain cases, patients may also experience systemic symptoms and multi-organ damage, raising the risk of B-cell non-Hodgkin lymphoma [1]. In China, the prevalence of pSS is 0.3–0.7% [2].

The precise origins of pSS remain elusive, but there are numerous study evidences indicating a reduction in T cells and excessive proliferation of B cells as the principal immunological abnormalities in pSS patients. The atypical proliferation of B cells into plasma cells results in the production of a multitude of immune complexes and autoantibodies, which is considered a pivotal step in the pathogenesis. The detection of serological autoantibodies is a vital aspect of pSS diagnosis. However, presently, only anti-SSA and anti-SSB antibodies are included in the diagnostic criteria, and their sensitivity and specificity are relatively low, falling short of clinical requirements [3]. Due to the intricate nature of pSS pathogenesis, diagnostic criteria continue to evolve, yet a significant number of patients remain undiagnosed and untreated, with an average diagnostic delay of 7 years [4]. Consequently, there is a pressing need for new autoantibodies as diagnostic markers to enhance the diagnostic rate.

Recent years have witnessed a surge in studies exploring novel antibodies associated with pSS, with particular attention given to antibodies targeting aquaporin, which are regarded as some of the most promising serological markers [5]. Aquaporins (AQPs) constitute a family of small transmembrane proteins, each weighing approximately 28 kDa, responsible for facilitating water transport across cell membranes in all living organisms. These AQPs are broadly expressed in exocrine glands, with 13 mammalian AQPs identified so far. Among them, AQP5 is recognized for its significant role in salivary secretion [6]. Although AQPs have a critical role in pSS pathogenesis, the presence of anti-AQP antibodies holds substantial diagnostic potential for pSS.

Antibodies against AQP1 (anti-AQP1) and AQP4 (anti-AQP4) have consistently been identified in the sera of patients afflicted with neuromyelitis optica (NMO). Anti-AQP4 is specifically discerned in NMO patients, and the presence of anti-AQP4 IgG in serum is regarded as the benchmark for distinguishing NMO from multiple sclerosis [7]. NMO spectrum disorders are occasionally associated with other autoimmune conditions, including SS [8]. Therefore, we postulated that individuals with SS may harbor autoantibodies targeting AQP. Recent investigations have also documented the detection of multiple antibodies against AQP in individuals grappling with SS. Furthermore, patients bearing AQP autoantibodies exhibited more pronounced symptoms of dry eye, suggesting a potential pathogenic role for these antibodies [9]. The anti-AQP1 was ascertained in 27.7% of SS patients and was absent in the control group [10]. Additionally, autoantibody against AQP5 (anti-AQP5) was observed in a subset of SS patients and associated with markedly reduced basal salivary secretion rates, demonstrating a sensitivity of 0.73 and specificity of 0.68 [11]. Although several studies have reported the presence of AQP1 and AQP5 autoantibodies in pSS patients, these investigations encompassed relatively small sample sizes, and the conclusions across various studies diverged. Therefore, to uncover potential novel diagnostic biomarkers for pSS, we undertook an exploration of the diagnostic utility of anti-AQP1and anti-AQP5 in the context of pSS.

## Materials and methods

### Clinical samples

A case–control study was conducted, and all subjects were drawn from the Department of Rheumatology and Immunology at the First Affiliated Hospital of Army Medical University between September 2021 and January 2022. The experimental group consisted of patients diagnosed with pSS who met the 2016 American College of Rheumatology (ACR)/European League against Rheumatism (EULAR) classification criteria for pSS [12]. They were excluded from other connective tissue diseases, acute and chronic infections, immunodeficiency disorders, and malignant tumors. The control group was comprised of patients with other connective tissue diseases, excluding Sjögren’s syndrome, who met the corresponding international diagnostic criteria. Healthy controls were recruited from the physical examination center, encompassing adults aged 18 to 65. Through the consecutive collection of data from approximately 30,000 patients who visited the clinic, a total of 163 subjects were ultimately included in the study, with 63 pSS patients and 61 patients with other connective tissue diseases were from our outpatient clinic and inpatient wards, as well as 39 healthy individuals from the physical examination center. The demographic, laboratory, and clinical characteristics of the subjects are displayed in Table 1. The study was granted approval by the Ethics Committee of the First Affiliated Hospital (Southwest Hospital) of Army Medical University (No: (A) KY2021075), and all participants were required to provide written informed consent voluntarily.

**Table 1:** Demographic and laboratory characteristics of the subjects

	pSS (*n* = 63)	CTD (*n* = 61)	HC (*n* = 39)
Sex (female), *n* (%)	58 (92.06)	52 (85.25)	29 (74.36)
Age (years), mean ± SD	47.87 ± 10.96	49.26 ± 12.82	41.82 ± 11.66
Time (years), M (P_25_, P_75_)	3.00 (2.00, 8.00)	3.00 (1.60, 4.00)	0 (0)
Anti-SSA + , *n* (%)	60 (95.24)	25 (40.98)	NT
Anti-SSB + , *n* (%)	36 (57.14)	5 (8.20)	NT
ANA + , *n* (%)	50 (79.37)	38 (62.30)	NT
RF + , *n* (%)	27 (42.86)	16 (26.23)	NT
Anti-ds-DNA + , *n* (%)	23 (36.51)	17 (27.87)	NT
complement 3, M (P_25_, P_75_)	0.78 (0.67, 0.88)	0.82 (0.75, 0.91)	NT
Lym count, M (P_25_, P_75_)	1.32 (0.83, 1.84)	1.40 (1.18, 1.76)	NT

*NT* not test

### Enzyme-linked immunosorbent assay (ELISA) and other experimental methods

There is no commercially available ELISA kit for human anti-AQP1 and AQP5 autoantibodies on the market. The ELISA kits used in this study were prepared by Jiangsu Jingmei Biological Technology Co. Ltd, China. Ninety-six-well plates were coated by overnight incubation at 4 °C with specific protein of Human AQP1 (No: Ag14093) or Human AQP5 (No: Ag14514. Wuhan Sanying Biotechnology Co., Ltd.) in 100 μl of 0.01 M carbonate buffer, pH 9.6, per well at a concentration of 2 μg/ml, respectively. After washed three times with phosphate buffer saline (PBS)/0.05% Tween 20, 150 μl blocking buffer (4% bovine serum albumin in PBS) was incubated for 2 h at room temperature. The pre-coated plates were successfully prepared after washed and air-dried for 6 h at room temperature. The levels of anti-AQP1 and anti-AQP5 IgG antibodies in the peripheral blood serum of the study participants were assessed through ELISA according to the manufacturer’s protocol. All measurements were performed in duplicated wells and the standard curve equations were built to calculate the corresponding concentrations (ng/ml) of anti-AQP1 and anti-AQP5 in the serum respectively according to OD values at wavelength 450 nm.

Additionally, clinical data including antinuclear antibodies (ANA), anti-SSA antibody, anti-SSB antibody, double strand (ds)-DNA, rheumatoid factor (RF), complement 3 levels, and lymphocyte count were collected from patients. ANA was detected using indirect immunofluorescence method; anti-SSA antibody, anti-SSB antibody, and ds-DNA were detected using immunoblotting method; RF was detected by immunoturbidimetry; complement 3 level was measured using immunochemistry method; lymphocyte count was determined using laser scattering method. All test data underwent statistical analysis according to corresponding standard methods conducted by the First Affiliated Hospital (Southwest Hospital) of Army Medical University.

### EULAR primary Sjögren’s syndrome disease activity index (ESSDAI)

For each patient with pSS, disease activity was assessed using the EULAR primary Sjögren’s syndrome disease activity index (ESSDAI), which is widely accepted in clinical practice as the gold standard for measuring the disease activity of Sjögren’s syndrome. ESSDAI encompasses 12 affected sites and evaluates systemic symptoms (such as fever and changes in body weight), lymph nodes, glands, joints, skin, lungs, kidneys, muscles, peripheral nerves, central nerves, hematological systems, and serological changes. Each affected site was graded for disease activity (inactive, mild, moderate, or severe). The final total score was calculated based on the weight of the affected site and the level of disease activity. ESSDAI scores less than 5 were defined as indicating low activity, scores between 5 and 13 as representing moderate activity, and scores equal to or greater than 14 were designated as indicating high activity [13].

### Statistical analysis

Statistical analysis was performed using SPSS 26.0 statistical software. Normally distributed measurement data were expressed as means and were subjected to analysis of variance for intergroup comparisons. Non-normally distributed data were presented as M (P25, P75) and were analyzed between groups using the Kruskal–Wallis H test. Measurement data correlation analysis was performed using Spearman correlation. For the assessment of clinical diagnostic value, receiver operating characteristic curves (ROC curves) were constructed. Key parameters, including the optimal cut-off value, sensitivity, specificity, area under the curve (AUC), and Youden index, were calculated. The statistical significance was defined as *P* < 0.05.

PASS 15.0.5 was used to calculate the sample size of this study. Select tests for one-sample Sensitivity and specificity procedure in PASS. (The relevant parameters are as follows: Solve For = Sample Size (Sensitivity), Power = 0.9, Alpha = 0.05, P (Prevalence) = 0.003 and 0.007 [2], Se0 (Null Sensitivity) = 0.73 [11], Se1 (Alternative Sensitivity) = 0.95, Sp0 (Null Specificity) = 0.68 [11], Sp1 (Alternative Specificity) = 0.70, H1 (Sensitivity): Se ≠ Se0, H1 (Specificity): Sp ≠ Sp0.)

## Results

### Baseline characteristics

A total of 163 subjects were divided into the pSS group (*n* = 63), other connective tissue diseases (CTD) group (*n* = 61), and healthy control (HC) group (*n* = 39). As calculated by PASS software, we enrolled an adequate sample size. The CTD group included cases of rheumatoid arthritis (*n* = 24), systemic lupus erythematosus (*n* = 13), undifferentiated connective tissue disease (*n* = 8), systemic sclerosis (*n* = 7), mixed connective tissue disease (*n* = 3), polymyositis (*n* = 2), dermatomyositis (*n* = 3), and IgG4-related disease (*n* = 1). The demographic, clinical, and laboratory characteristics of the study subjects are presented in Table 1.

### Analysis of anti-AQP1 and anti-AQP5 autoantibodies levels

**Fig. 1:**
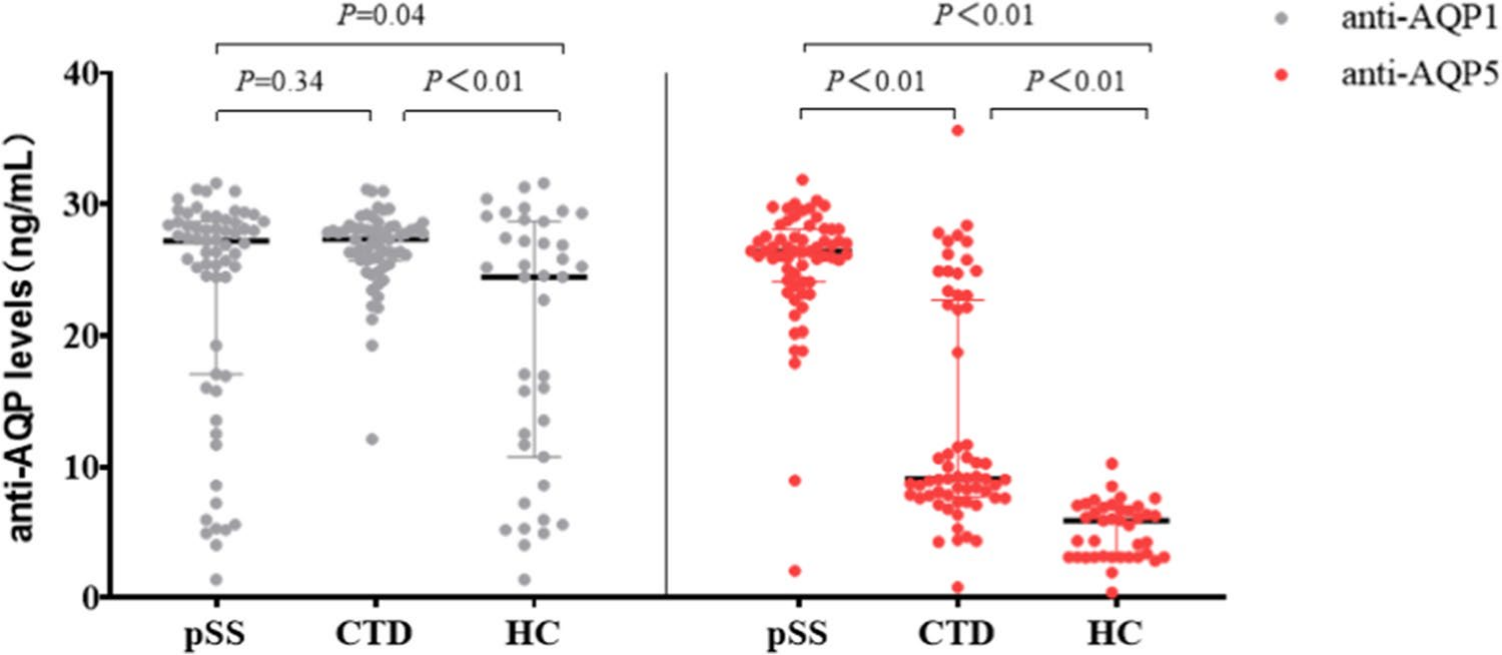
Subject serum concentrations of anti-AQP1 and anti-AQP5. CTD, connective tissue diseases; HC, healthy control. *P < 0.05, statistically significant difference

In the pSS group (*n* = 63), CTD group (*n* = 61), and HC group (*n* = 39), the concentrations of serum anti-AQP1 and anti-AQP5 were determined by ELISA. The results revealed that the concentrations of anti-AQP1 in the three groups were 27.20 (17.07, 28.68) ng/ml, 27.34 (25.70, 28.18) ng/ml, and 24.44 (10.77, 28.68) ng/ml, respectively. It was observed that the pSS group had higher levels than the HC group (*P* = 0.04), and the CTD group displayed higher levels than the HC group (*P* < 0.01). No statistically significant difference was found in concentration between the pSS group and the CTD group (*P* = 0.34) (Table 2, Fig. 1). The concentrations of anti-AQP5 in the three groups were 26.42 (24.07, 28.08) ng/ml, 9.10 (7.73, 22.70) ng/ml, and 5.93 (3.13, 6.90) ng/ml, respectively. The results indicated that the pSS group exhibited higher levels than the CTD group and the HC group (*P* < 0.01) (Table 2, Fig. 1).

**Table 2:** Comparison of serum concentrations of anti-AQP in the subjects

Group	*n*	anti-AQP1 (ng/ml)	anti-AQP5 (ng/ml)
pSS	63	27.20 (17.07, 28.68)	26.42 (24.07, 28.08)
CTD	61	27.34 (25.70, 28.18)	9.10 (7.73, 22.70)
HC	39	24.44 (10.77, 28.68)	5.93 (3.13, 6.90)
χ^2^	-	8.21	102.27
*P*	-	0.02	< 0.01

### ROC curve analysis of anti-AQP1 and anti-AQP5 levels

**Fig. 2:**
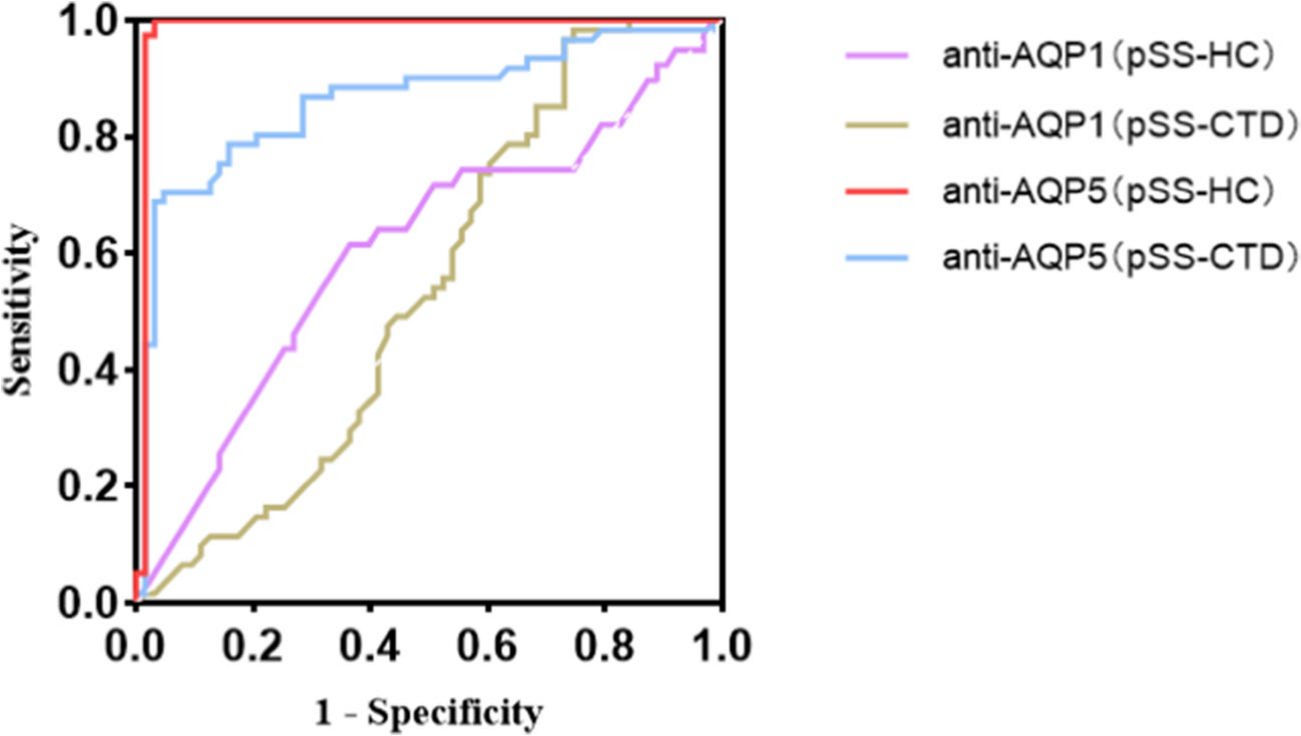
ROC curves of anti-AQP1 and anti-AQP5. HC, healthy control; CTD, connective tissue diseases; pSS-HC, pSS group as the case group, HC as control group; pSS-CTD, pSS group as the case group, CTD as control group. *P < 0.05, statistically significant difference

To further investigate the clinical diagnostic significance of anti-AQP1 and anti-AQP5 in pSS patients, we constructed ROC curves. The case group comprised 63 pSS patients, while the control groups included 39 healthy individuals and 61 CTD patients. We calculated the AUC, Youden index, cut-off value, sensitivity, specificity, and likelihood ratio of anti-AQP1 and anti-AQP5 for discriminating between pSS and non-pSS. The results are presented in Table 3 and Fig. 2. The analysis showed that anti-AQP1 had no diagnostic value for pSS (*P* > 0.05). In 39 healthy controls, all anti-AQP5 tests were below the cut-off value (14.10 ng/ml), with an AUC of 0.98 (95% CI 0.96–1.00), a sensitivity of 0.97 (95% CI 0.89–0.99), and a specificity of 1.00 (95% CI 0.91–1.00), the positive likelihood ratio was infinity, and the negative likelihood ratio was 0.03 (95% CI 0.03–0.12) (*P* < 0.05); when the CTD group was used as the control group, the AUC was 0.86 (95% CI 0.80–0.93), the cut-off value was 18.79 ng/ml, the sensitivity was 0.95 (95% CI 0.87–0.99), the specificity was 0.70 (95% CI 0.58–0.84), the positive likelihood ratio was 3.23 (95% CI 2.18–4.78), and the negative likelihood ratio was 0.07 (95% CI 0.02–0.21) (*P* < 0.05) (Table 3, Fig. 2).

**Table 3:** ROC curve analysis results of anti-AQP in the subjects

ROC curve	AUC (95% CI)	*P*	Youden index	Cut-off value (ng/mL)	Sensitivity (95% CI)	Specificity (95% CI)	Positive likelihood ratio (95% CI)	Negative likelihood ratio (95% CI)
Anti-AQP1(pSS-HC)	0.61 (0.49–0.72)	0.07	0.25	25.40	0.63 (0.51–0.74)	0.62 (0.46–0.75)	1.65 (1.06–2.56)	0.59 (0.42–0.85)
Anti-AQP1(pSS-CTD)	0.46 (0.36–0.56)	0.42	0.43	28.66	0.25 (0.16–0.38)	0.84 (0.71–0.91)	1.54 (0.76–3.14)	0.89 (0.77–1.04)
Anti-AQP5(pSS-HC)	0.98 (0.96–1.00)	0.0001	0.97	14.10	0.97 (0.89–0.99)	1.00 (0.91–1.00)	Infinity	0.03 (0.01–0.12)
Anti-AQP5(pSS-CTD)	0.86 (0.80–0.93)	0.0001	0.66	18.79	0.95 (0.87–0.99)	0.70 (0.58–0.84)	3.23 (2.18–4.78)	0.07 (0.02–0.21)

### Correlation analysis of anti-AQP1 and anti-AQP5 levels with ESSDAI

Among the 63 pSS patients included in this study, 8 were classified as low activity, 37 as moderate activity, and 18 as high activity. The serum levels of anti-AQP1 autoantibodies in pSS patients with low, moderate, and high activity were (19.93 ± 10.93) ng/ml, (25.90 ± 9.94) ng/ml, and (20.80 ± 9.75) ng/ml, respectively. No significant difference was observed among the three groups (*P* > 0.05). Similarly, the concentrations of anti-AQP5 in the three groups were (26.01 ± 2.92) ng/ml, (27.14 ± 8.24) ng/ml, and (23.90 ± 5.67) ng/ml, with no significant difference among the three groups (*P* > 0.05) (Table 4).

**Table 4:** Comparison of anti-AQP levels in pSS patients with different disease activity

ESSDAI	*n*	Anti-AQP1 (ng/ml)	Anti-AQP5 (ng/ml)
Low activity	8	19.93 ± 10.93	26.01 ± 2.92
Moderate activity	37	25.90 ± 9.94	27.14 ± 8.24
High activity	18	20.80 ± 9.75	23.90 ± 5.67
F	-	2.22	1.25
*P*	-	0.12	0.30

*P < 0.05, statistically significant difference

### Correlation analysis between anti-AQP5 level and various clinical indicators

In a total of 63 pSS patients, there was no significant correlation observed between anti-AQP5 (26.42 (24.07, 28.08) ng/ml) levels and anti-SSA (200.00 (94.90, 200.00) IU/ml), anti-SSB (101.00 (6.47, 400.00) IU/ml), ANA (320.00 (320.00, 1000.00)), RF (20.00 (20.00, 51.50) IU/ml), anti-ds-DNA (25.00 (25.00, 42.00) IU/ml), complement 3 (0.78 (0.67, 0.88) g/l), or lymphocyte count (1.32 (0.83, 1.84)) levels (*P* > 0.05). Furthermore, salivary gland flow rate was measured in a subset of 30 pSS patients (≤ 1.5 ml/15 min classified as reduced; > 1.5 ml/15 min classified as normal), among whom 8 exhibited normal salivary gland flow rate while 22 had reduced flow rate. Importantly, there was no statistically significant association found between anti-AQP5 (26.59 (25.58, 18.18) ng/ml) and salivary gland flow rate (1.00 (0.30, 1.80) ml/min) levels (*P* > 0.05).

## Discussion

Currently, the diagnosis of pSS relies on a comprehensive assessment that considers both subjective symptoms related to the mouth and eyes, as well as objective evaluations of the lacrimal and salivary glands, serological markers, and histopathological findings. Unfortunately, the clinical use of eye, salivary gland, and tissue biopsies is inconsistent, which may result in the oversight or misdiagnosis of pSS patients [14]. Additionally, there is a worldwide lack of standardized classification criteria for Sjögren’s syndrome, especially when patients present with only nonspecific symptoms, leading to insufficient diagnostic criteria and delayed diagnosis [15]. The production of autoantibodies and the formation of immune complexes play a pivotal role in the pathogenesis of pSS. While SSA and SSB autoantibodies are included in the diagnostic criteria, SSB autoantibody exhibits low sensitivity, and there are no significant differences in clinical features between pSS patients with positive and negative SSB autoantibodies [16]. Moreover, SSA autoantibodies are only present in 50–70% of SS patients [3]. In the 2016 ACR/EULAR pSS classification criteria [12], only SSA autoantibody are considered, but it is evident that SSA autoantibody alone, with their low sensitivity, cannot serve as adequate serological diagnostic markers to meet clinical needs. Therefore, the quest for new autoantibody diagnostic markers is of great significance.

Although AQP1-deficient mice do not manifest defects in salivary volume or composition [17], the expression of AQP1 is downregulated in the salivary glands of SS patients. Furthermore, B-cell depletion with rituximab increased AQP1 expression in myoepithelial cells and salivary flow rate in SS patients [18]. Additionally, the adenoviral-mediated transfer of AQP1 cDNA significantly enhanced salivary flow rates in irradiated rats, human subjects with radiation-induced salivary hypo-function, and a murine model of SS [19]. Our results showed that the level of anti-AQP1 in the pSS group was higher than in the HC group. However, the AUC was very low and was not correlated with the salivary flow rate and ESSDAI, indicating that anti-AQP1 does not prove useful as diagnostic markers.

Numerous studies have affirmed the pivotal role of AQP5 in salivary fluid secretion [20]. AQP5 knockout mice exhibited a reduction of over 60% in pilocarpine-stimulated saliva production. Saliva from these knockout mice was hypertonic (420 mosM) and significantly more viscous when compared to saliva from wild-type mice [21]. Aberrant expression and localization of AQP5 have been closely linked to the pathogenesis of Sjögren’s syndrome. Immunolabeling of AQP5 in salivary gland sections from various animal models of SS and some SS patients revealed anomalous expression and/or localization in acinar cells, characterized by a predominant basolateral and/or intracellular localization, rather than the typical apical membrane localization [22]. Other studies have suggested that the deviant AQP5 localization in SS mouse models is likely due to the presence of inflammatory infiltrates [23]. The study demonstrated a significant elevation in serum concentrations of anti-AQP5 in pSS samples compared to control samples, accompanied by increased sensitivity (0.95) and specificity (0.70). This emphasized the close relationship between anti-AQP and the pathogenesis of pSS. Furthermore, the study reveals that anti-AQP5 possess higher sensitivity compared to anti-SSA antibody, potentially compensating for the limited detection rate of anti-SSA antibody in SS patients. Combining the use of anti-AQP5 could enhance the diagnostic rate of SS. Notably, there was no statistical correlation observed between anti-AQP5 and the ESSDAI disease activity score, salivary gland flow rate, anti-SSA, anti-SSB, ANA, rheumatoid factor, anti-ds-DNA, complement 3, and lymphocyte count in this study, indicating that anti-AQP5 levels are independent of disease activity. However, our research results differ from previous studies, which found an association between anti-AQP5 levels and decreased salivary flow rate in pSS patients [24]. This discrepancy may be related to the relatively small number of pSS patients included in our salivary flow rate examination, and further exploration is needed for validation.

At present, there are only several small sample studies reporting the clinical significance of anti-AQP5 in pSS patients. A Korean study indicated that 73.2% of SS patients and 32.1% of healthy controls were AQP5 IgG positive, yielding a sensitivity of 0.73 and specificity of 0.68 [11]. This was replicated in a non-Korean cohort with a sensitivity of 0.61 and specificity of 0.77 [24]. Our results of higher sensitivity of anti-AQP5 might be associated with our enrollment of 95.24% anti-SSA positive in patients with pSS.

In conclusion, serum anti-AQP5 levels significantly increased in pSS patients, with a sensitivity of 0.95 and a specificity of 0.70, indicating that anti-AQP5 is a novel diagnostic biomarker for pSS. Further prospective studies in large-size multicenter cohorts and diverse experimental methods are needed to validate the diagnostic advantage of the AQP5 antibody for SS patients in the future.
